# Timing of Single-Neuron and Local Field Potential Responses in the Human Medial Temporal Lobe

**DOI:** 10.1016/j.cub.2013.12.004

**Published:** 2014-02-03

**Authors:** Hernan Gonzalo Rey, Itzhak Fried, Rodrigo Quian Quiroga

**Affiliations:** 1 Centre for Systems Neuroscience, University of Leicester, Leicester LE1 7QR, UK; 2 Department of Neurosurgery and Semel Institute for Neuroscience and Human Behavior, University of California, Los Angeles, Los Angeles, CA 90095-7039, USA; 3 Functional Neurosurgery Unit, Tel Aviv Medical Center and Sackler Faculty of Medicine, Tel Aviv University, Tel Aviv 64239, Israel

## Abstract

The relationship between the firing of single cells and local field potentials (LFPs) has received increasing attention, with studies in animals [1, 2, 3, 4, 5, 6, 7, 8, 9, 10, 11] and humans [12, 13, 14]. Recordings in the human medial temporal lobe (MTL) have demonstrated the existence of neurons with selective and invariant responses [15], with a relatively late but precise response onset around 300 ms after stimulus presentation [16, 17, 18] and firing only upon conscious recognition of the stimulus [19]. This represents a much later onset than expected from direct projections from inferotemporal cortex [16, 18]. The neural mechanisms underlying this onset remain unclear. To address this issue, we performed a joint analysis of single-cell and LFP responses during a visual recognition task. Single-neuron responses were preceded by a global LFP deflection in the theta range. In addition, there was a local and stimulus-specific increase in the single-trial gamma power. These LFP responses correlated with conscious recognition. The timing of the neurons’ firing was phase locked to these LFP responses. We propose that whereas the gamma phase locking reflects the activation of local networks encoding particular recognized stimuli, the theta phase locking reflects a global activation that provides a temporal window for processing consciously perceived stimuli in the MTL.

## Highlights

• Global theta LFP increases immediately precede MTL single-cell responses
• Gamma power reflects activations of local networks encoding specific stimuli
• The timing of the neurons’ firing is phase locked to LFP responses
• LFP responses give a temporal window for processing consciously perceived stimuli

## Teaser

Rey et al. show that, in human medial temporal lobe (MTL), single-cell responses triggered by consciously perceived stimuli are locked to global theta and local gamma LFP responses; the latter reflects local activations, but the former shortly precedes the spike responses and may provide a window for stimulus processing in the MTL.

## Results

We recorded single-neuron and local field potential (LFP) activity during twelve sessions in five patients with pharmacologically intractable epilepsy, who were implanted with intracranial electrodes for clinical reasons. All patients gave their written informed consent to participate in the study, which conformed to the guidelines of the Medical Institutional Review Board at University of California, Los Angeles. Subjects were shown a sequence of briefly presented pictures (followed by a mask) and had to report whether or not they recognized the picture [19] (see Supplemental Experimental Procedures and Figure S1 available online for behavioral results). Altogether, we found 76 significant responses (see Supplemental Experimental Procedures) (1 from amygdala, 35 from entorhinal cortex, and 40 from hippocampus), coming from 41 different units in 37 different channels.

**Figure 1:**
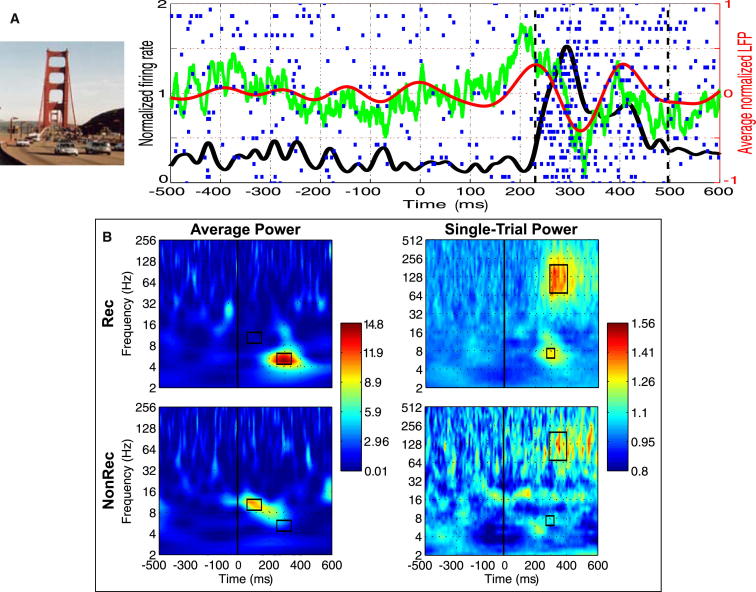
LFP Power in Recognized and Nonrecognized Trials (A) Example of a neuron in the left entorhinal cortex that responded to a picture of the Golden Gate Bridge. The raster plot, the instantaneous firing rate (solid black line), onset and offset of the spiking response (dashed vertical lines), raw average local field potential (LFP, green line), and average LFP in the theta band (4–8 Hz; red line) are shown. Only the 29 recognized trials were used for computing firing rate and average LFP. More examples can be found in Figure S3. (B) Left: time-frequency plot of the average LFP (evoked) power for recognized (Rec) and nonrecognized (NonRec) trials. The regions of interest (ROIs) used for statistical comparisons are indicated by black rectangles (see Supplemental Experimental Procedures). There is a significant increase in the theta band for Rec trials (p = 2 × 10^−3^) and in the alpha band for NonRec trials (p = 0.02). Right: grand median time-frequency plot of the single-trial LFP power for Rec and NonRec trials. There is a significant increase in theta (p < 10^−12^) and gamma (p < 10^−23^) bands for Rec trials, but not for NonRec trials (p > 0.08).

**Figure 2:**
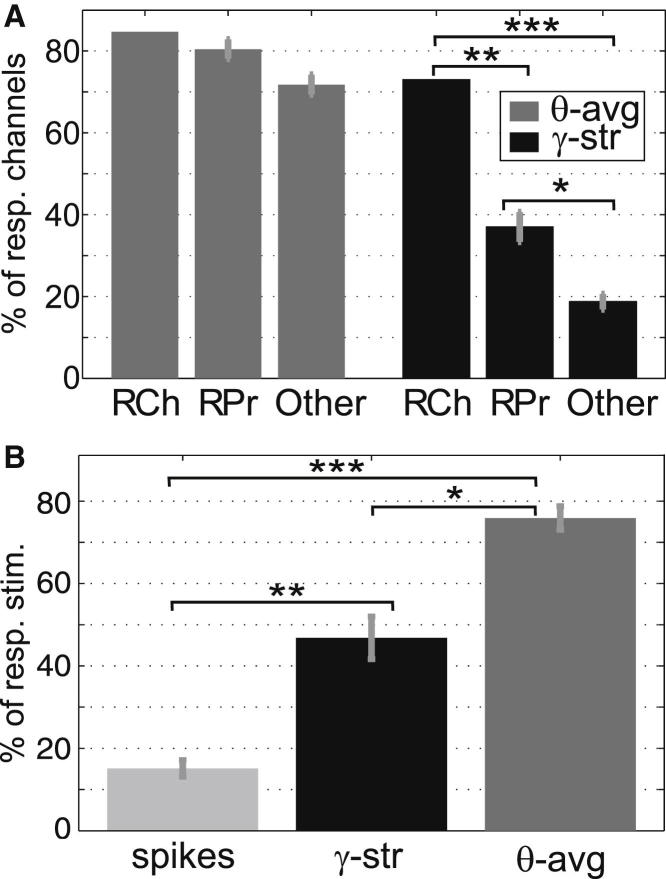
LFP Power Selectivity (A) Probability of LFP responses in the channels with unit responses (RCh), in other channels from the same probe (RPr), and in channels from other probes (Other). Whereas for the average theta power the probability of responses was statistically the same for all channels (p > 0.18), for single-trial gamma power it decreased significantly for channels further away from the sites with the unit responses (^∗^p < 10^−5^, ^∗∗^p < 10^−8^, ^∗∗∗^p < 10^−28^). (B) The percentage of stimuli eliciting unit, gamma, or theta responses was significantly different (Kruskal-Wallis test, p < 10^−13^). Post hoc analysis showed significant differences for all conditions (^∗^p < 10^−4^, ^∗∗^p < 10^−5^, ^∗∗∗^p < 10^−11^). Error bars represent SEM.

We then extracted the LFPs in those channels for each trial, separating between recognized and nonrecognized stimuli for further analysis. Figure 1A shows an exemplary response of a neuron in the left entorhinal cortex that fired selectively to a picture of the Golden Gate Bridge. This activation was correlated with an evoked response in the theta band (4–8 Hz), a pattern that was common for most responses (see leftmost column of Figure 2A and Figure S3 for more examples). In fact, the time-frequency plot of the evoked response for the recognized trials (Figure 1B, left) shows a significant increase with respect to baseline (p = 2 × 10^−3^) in the theta band around 300 ms after stimulus onset. In contrast, for the nonrecognized trials the theta-evoked response was not present (p = 0.23 compared to baseline) and instead, there was a less pronounced but significant increase (p = 0.03) in the alpha band (∼10 Hz), starting at 60 ms after stimulus onset, which was not present in the recognized trials (p = 0.4). A direct comparison between the recognized and nonrecognized trials showed that both the theta increase for the recognized trials and the alpha increase for the nonrecognized ones were significantly larger than for the other condition (p < 10^−10^ in both cases).

Given that single-trial responses may cancel out when averaging across trials, we also studied the power of the single-trial LFP responses (Figure 1B, right) instead of calculating the power after averaging, as with the evoked responses. For the recognized trials, there was an increase in single-trial power with respect to baseline in the theta and high-gamma (70–200 Hz) bands (p < 10^−12^ and p < 10^−23^, respectively), and no significant increases were found for the nonrecognized trials (p = 0.5 and p = 0.08 for the theta and gamma bands, respectively). Comparing both conditions, the gamma single-trial power for the recognized trials was significantly higher than for the nonrecognized ones (p < 10^−4^), but for the theta region this difference showed only a tendency that did not reach significance (p = 0.08). Altogether, the unit responses upon picture recognition were correlated with an increase in the (evoked) theta and the (single-trial) gamma power in the LFP signals.

### Selectivity Analysis

Next, we studied the probability of occurrence and degree of spatial localization of these LFP responses (Figure 2A). Almost 85% of the channels exhibiting a unit response also showed a theta LFP response. These responses were not spatially localized, as the percentage of responsive channels showing a theta response was not significantly different for other nearby channels in the same probe (see Supplemental Experimental Procedures) or for channels further away in other probes (p > 0.18). In contrast, the probability of finding gamma responses decreased significantly for more distant channels (p < 10^−5^), going from 73% in the channels with unit responses down to 19% for distant channels. Complementing these results, we studied the selectivity of the spiking and LFP responses to the different (recognized) stimuli (Figure 2B). The spiking responses were the most selective ones (15%) (see Figure S2 for more results on the selectivity of the spiking responses), followed by the single-trial gamma responses (47%), whereas the theta-evoked responses were not selective at all, as they were triggered by 76% of the stimuli. The difference in selectivity between these three responses was highly significant (p < 10^−13^). In summary, the selectivity analysis shows that the theta LFP response was global and present for most (recognized) stimuli, whereas the gamma LFP response was local and more stimulus specific.

### Timing of the Spike and LFP Responses

**Figure 3:**
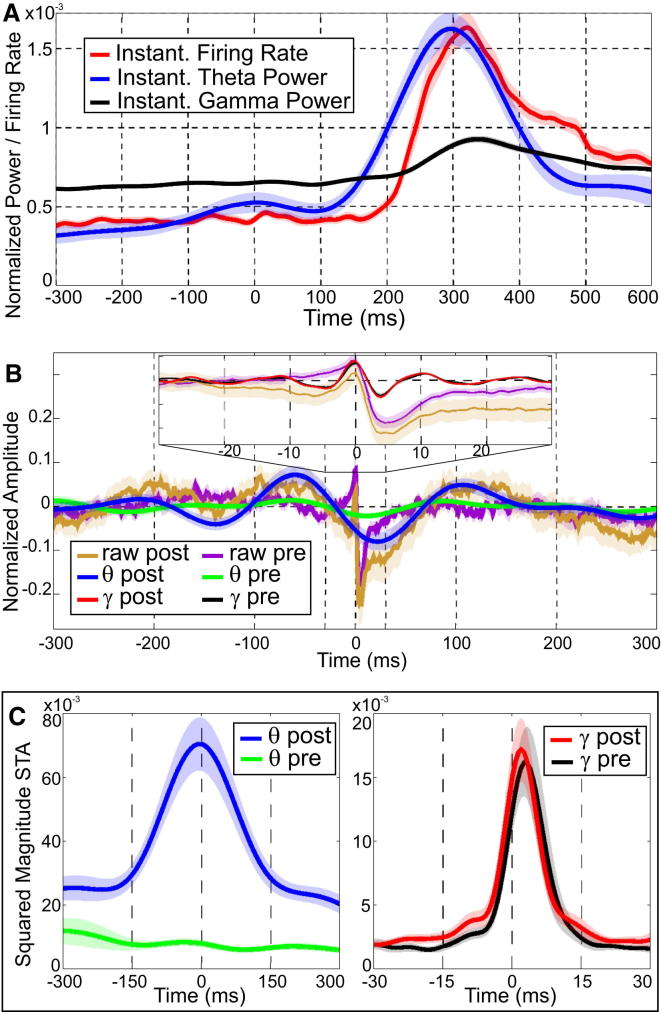
Instantaneous Power and Spike-Triggered Average (A) Grand average of the instantaneous firing rate, theta-evoked power, and single-trial gamma power. The increase in theta power precedes the increase in firing rate by 50–100 ms, whereas the gamma power has approximately the same onset as the instantaneous firing rate. (B) Grand average spike-triggered average (STA) in the pre- and poststimulus epochs (see Supplemental Experimental Procedures). Raw and theta-filtered data are shown in the plot; raw and gamma-filtered data are shown in the inset. (C) Grand average of the square magnitude envelope of the STAs computed in the same epochs and bands as in (B). The shaded area indicates SEM. In (A), time is measured with respect to stimulus onset; in (B) and (C), time is measured with respect to the spike occurrence.

The distribution of the single-unit response onsets had a mean of 260 ms (median 246 ms; SD 56 ms; see Supplemental Experimental Procedures for definition of the spike response onset). For the channels with significant LFP and unit responses, we computed the instantaneous power using the squared magnitude of the Hilbert transform after band-pass filtering (see Supplemental Experimental Procedures). Figure 3A shows the normalized average responses, where we observe that the mean theta power activation shortly preceded the increase in firing rate by 50–100 ms, while increases in gamma power and firing rate appeared at approximately the same time. There were no differences in these dynamics when considering responses from different areas, i.e., entorhinal cortex versus hippocampus (data not shown).

To further explore the relationship between the unit and LFP activity, we also computed the spike-triggered averages (STAs) for each of the 76 unit responses. The grand average STA showed a strong locking of the spikes to the theta activity after the stimulus onset, but not during baseline (Figure 3B; see Figure S3 for examples from individual responses). This result was confirmed by averaging the squared magnitude of the Hilbert transform of each STA (Figure 3C, left), providing a more robust means of assessing the locking, regardless of the particular phases at which the spikes locked on individual responses. In contrast, locking with gamma activity was present both before and after stimulus presentation (inset in Figure 3B and Figure 3C, right).

**Figure 4:**
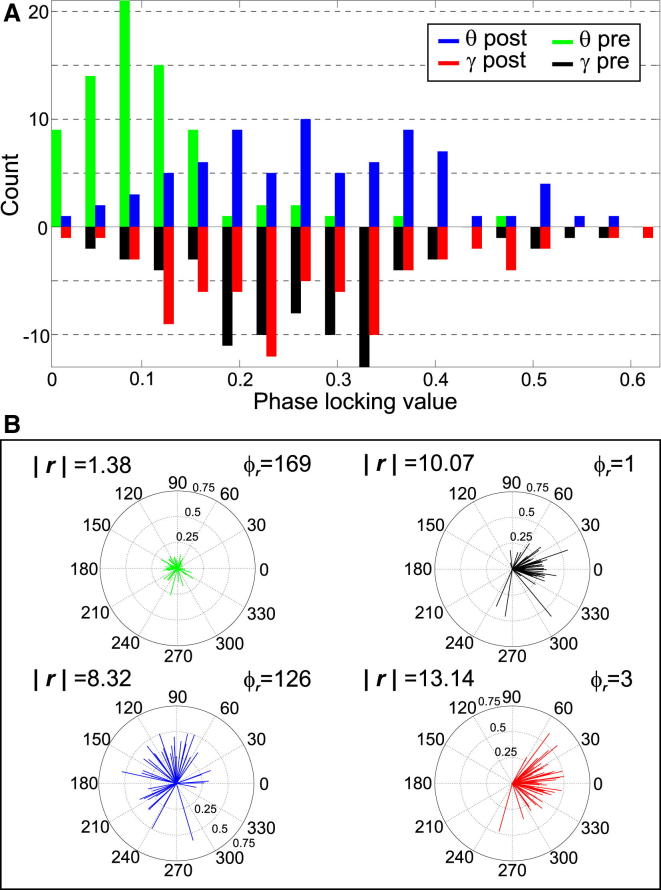
Phase-Locking Analysis (A) Histograms of the phase-locking values computed for the 76 spiking responses. There was a significant difference between pre- and poststimulus epochs for the theta band (p < 10^−15^), but not for the gamma band (p = 0.83). (B) Mean phase vectors for 47 and 48 significant responses (Rayleigh test, p < 0.05) in the gamma and theta bands, respectively. Color coding is the same as in (A). In each case, | *r* | and ϕ_*r*_ are the magnitude and phase of the sum of all individual mean phase vectors.

We further quantified these observations with a phase-locking analysis (see Supplemental Experimental Procedures and Figures S3 and S4). As shown in Figure 4A, the distribution of phase-locking values in the theta band for the 76 responses was significantly larger in the poststimulus than in the prestimulus epoch (rank-sum test, p < 10^−15^). In line with the STA results, this difference was not significant in the gamma band (rank-sum test, p = 0.83). Figure 4B shows the mean phases for the 47 of 76 and 48 of 76 responses that had a significant phase tuning (Rayleigh test, p < 0.05) in the poststimulus epoch for the gamma and theta bands, respectively (see Supplemental Experimental Procedures). For the theta band, there was a large tuning toward the upper left quadrant after stimulus presentation that was not present during baseline. In agreement with the STA results, the gamma phase tuning was similar in the response and baseline epochs, with an average phase around 0° (peak of the oscillation).

## Discussion

A classic approach in the study of visual awareness is to analyze behavioral and neural responses using paradigms in which visual stimuli are presented under conditions of ambiguous perception [20]. In particular, single-cell recordings in the monkey inferotemporal cortex (ITC) have shown differential responses in a shape discrimination task using backward masking [21] and with alternating percepts during binocular rivalry [22]. In humans, the study of visual awareness has been approached using different experimental techniques, and differential responses to recognized and nonrecognized stimuli have been described with fMRI recordings [23], scalp electroencephalography (EEG) [24, 25], and intracranial EEG [26]. With the same experiment of the current study, but reporting solely on the firing of single units, we have previously shown a strong correlation between spike responses and the subjective report of the subjects (stimulus recognized or not) in the human medial temporal lobe (MTL) [19]. These categorical all-or-none responses have been linked to conscious access [27], and others have argued that it might be merely the consequence of an actual conscious experience [28]. Adding to the finding of single-unit responses to consciously perceived images, we here describe an increase in the evoked theta and the single-trial gamma power, only for recognized trials. But beside the relation of these single-unit and LFP responses to visual awareness, the main objective of this work was to study the relationship between the LFP and spike responses in the human MTL to give insights into the timing of single units’ firing, and particularly the very late onset of the units’ responses.

Previous works in different species and areas have reported a pivotal role of LFP patterns in providing a precise timing of single-unit firing [1], and also for establishing the communication between different areas [29, 30]. For example, place cells in the rodent hippocampus [6] fire at precise phases of theta oscillations when the animal crosses the cell’s place field [31]. A similar temporal coding, given by the LFP phase of the neuron’s firing, was also reported to encode the position, trajectory, and heading of rats [8]. The precise firing of neurons in the rat entorhinal-hippocampal loop has been linked to the phase of the LFP in the theta [4] and gamma [2] bands. In monkeys, LFP activity in parietal cortex was coherent with the spiking activity during movement planning [9], and it was possible to predict time and direction of movement from these signals. Moreover, the LFP phase at the time of neuronal firing was also found to encode information in the monkey ITC [3] and superior temporal sulcus [11] during object perception tasks, and a similar LFP timing mechanism was shown in visual [5] and auditory [10] cortices during the perception of naturalistic stimuli. In addition, it has been shown in monkeys that spikes encoding two different objects in a short-term memory task were associated to different phases of an oscillation in the beta band (∼32 Hz) during the delay period [32].

Closer to our study, previous works in humans have also shown the influence of LFP activity in the neurons’ firing. In particular, it was found that the neurons’ firing was phase locked to oscillations in the theta and gamma bands in widespread brain regions, including the MTL [12]. Nir and colleagues [33] described a coupling between the activity of single neurons and the LFP in the gamma band in the auditory cortex during spontaneous activity and with sensory stimulation. In another work, Kraskov and colleagues [13] showed that phase locking of MTL neurons in the theta band and power increases in the gamma band were selective for categories of visual stimuli. Adding to these results, a remarkable correlation with behavior was reported by Rutishauser and colleagues [14], who showed that the coupling strength of the spikes with theta oscillations predicted successful memory formation. In line with this latter study, we describe here two distinct patterns of activations that are tightly correlated with the conscious recognition of images.

On the one hand, we found that conscious recognition elicited not only very selective single-cell responses in the human MTL but also a stimulus-specific increase of the single-trial power in the high-gamma band. Of note, it has been previously shown that increases in high-frequency power can be observed as a result of multiunit activity [7, 34]. However, in our case the high-gamma increase was localized in frequency—in particular, it decayed for higher frequencies—and consequently it cannot be attributed to a tail of the power spectrum generated by the multiunit firing. This activation was local—i.e., it was mainly present in the electrodes where we found single-unit responses—in agreement with spatially localized gamma responses reported in animal LFPs [2, 7, 35] as well as in human intracranial EEGs [26, 35, 36] and LFPs [33]. In general, increases in gamma power can be regarded as an index of neuronal synchronization reflecting local network computations [34], and therefore, given the large evidence supporting the role of the MTL in declarative memory [37] and considering the explicit and abstract representation given by human MTL neurons [15, 17, 18], we postulate that the single-trial gamma increases in our data reflect the activation of local cell assemblies that bring particular concepts into awareness for memory functions [18].

In addition, and more interestingly, we found that the MTL single-cell responses were preceded by a large deflection of the LFP in the theta range. As with the high-gamma responses described above, this activation was only present for the recognized stimuli, but in contrast to the gamma responses, it was not stimulus selective—i.e., it appeared for any stimulus, as long as it was recognized—and it was global—i.e., it was present in most MTL electrodes. In line with this finding, low-frequency activity has been linked to the activation of relatively large networks [1]. Furthermore, given the stereotyped LFP responses and the consistent onset of single-unit activations, we observed a strong phase locking in the theta band at the response time.

The latency of responses in different areas along the ventral visual pathway is determined by direct feedforward projections [38], culminating in activations at about 100 ms in monkey ITC [3, 38, 39] (see also Table 1 in [16]) and analogous structures in humans [40, 41]. There are direct projections from ITC to the MTL [42], and responses in monkeys performing visual/spatial recognition memory tasks show latencies between 100 and 200 ms in the hippocampus [43, 44] and entorhinal cortex [45]. However, responses in the human MTL, like the ones reported here, have a much later onset (∼300 ms) [15, 16, 18] than what would be expected from direct feedforward projections from ITC. We have previously argued that such a latency gap reflects the further processing of sensory stimuli in order to create a conceptual abstract representation that is used by the MTL for memory functions [18]. However, the neural mechanisms that account for such late but precise onset timing remained unclear. Within this context, the current data suggest that the theta LFP responses described above reflect an activation of inputs within the MTL [4] and/or from afferent activity from other cortical and subcortical networks [30] that provides a temporal window for triggering the single neuron firing upon picture recognition—a gateway for processing consciously perceived stimuli within the MTL. Given the proposed role of the MTL in combining information from different sensory modalities to create a unified percept [17, 18], the timing mechanism given by the theta LFP responses may be critical for synchronizing and combining multisensory information involving different processing times.

## Supplemental Information

Document S1. Figures S1–S4 and Supplemental Experimental Procedures
