# Supplementary material for: Timing of Single-Neuron and Local Field Potential Responses in the Human Medial Temporal Lobe
Source: Curr Biol. 2014 Feb 3;24(3):299–304. doi: 10.1016/j.cub.2013.12.004 (PMC3963414; doi:10.1016/j.cub.2013.12.004)
Supplement: Document S1. Figures S1–S4 and Supplemental Experimental Procedures [file mmc1.pdf]

Current Biology, Volume *24*

Supplemental Information

Timing of Single-Neuron and Local Field Potential Responses in the Human Medial Temporal Lobe

Hernan Gonzalo Rey, Itzhak Fried, and Rodrigo Quian Quiroga

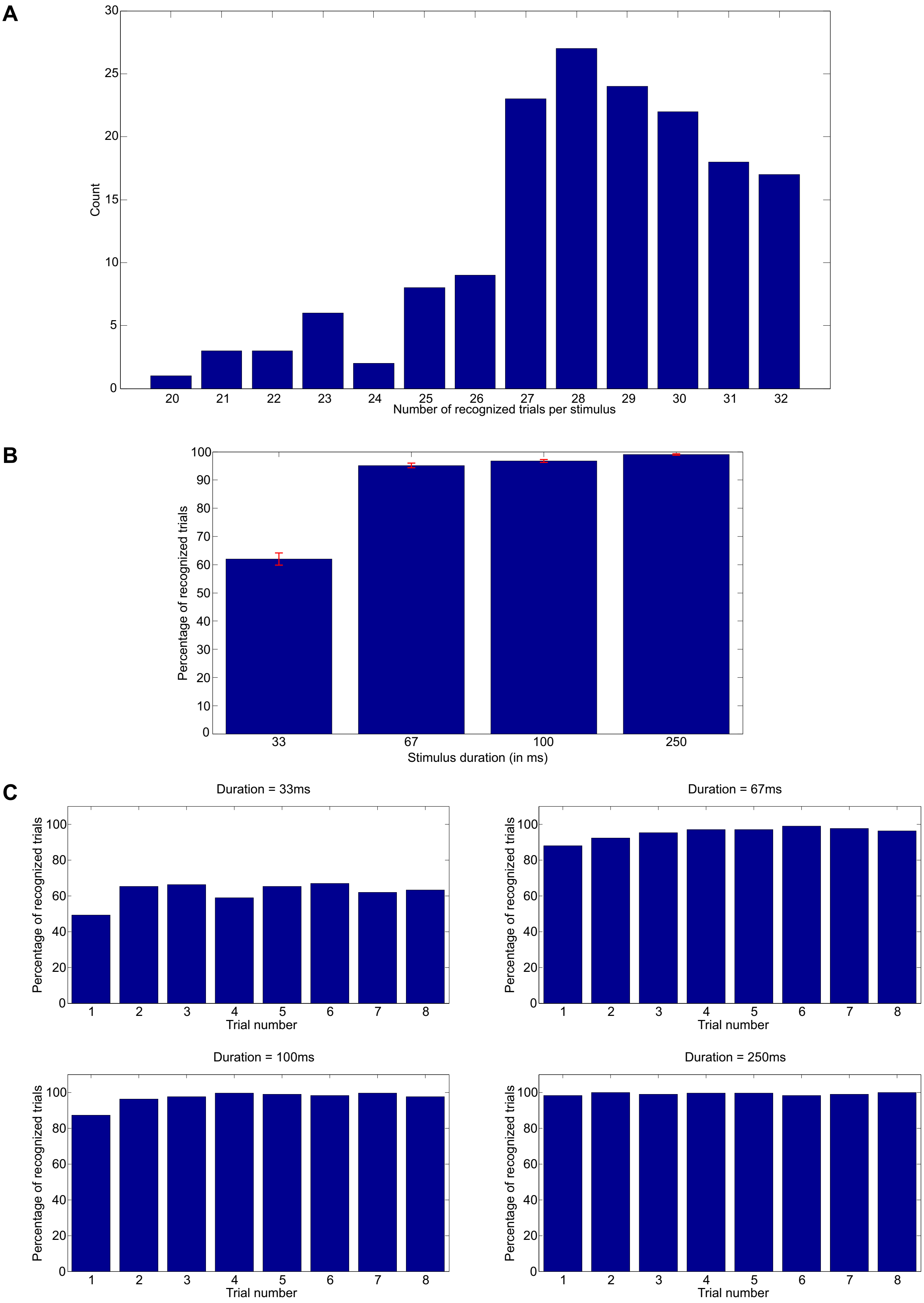
A
Count
Number of recognized trials per stimulus
B
Percentage of recognized trials
Stimulus duration (in ms)
C
Duration = 33ms
Duration = 67ms
Duration = 100ms
Duration = 250ms
Percentage of recognized trials
Trial number

Figure S1

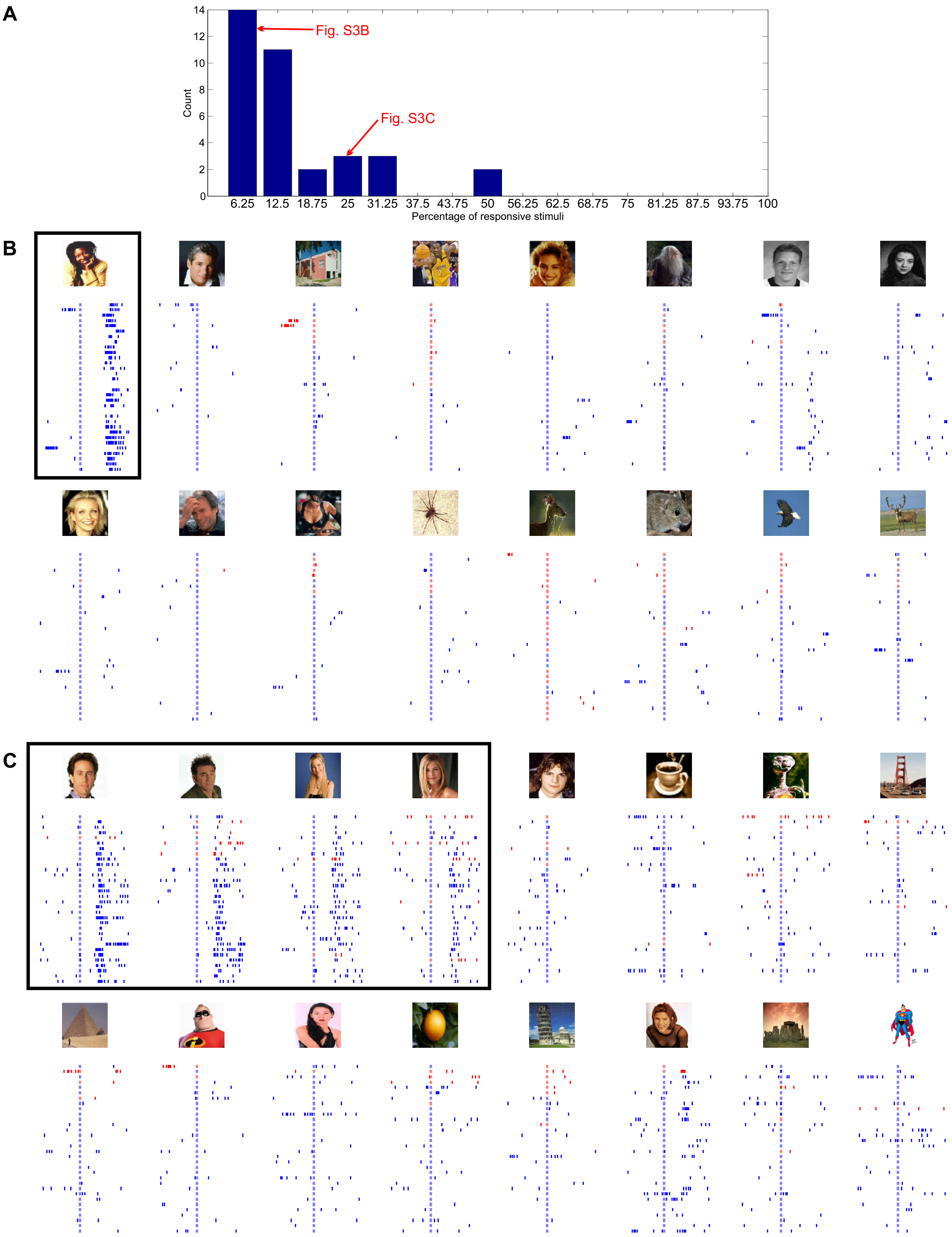
A
B
C
Count
Percentage of responsive stimuli
Fig. S3B
Fig. S3C
0
2
4
6
8
10
12
14
6.25
12.5
18.75
25
31.25
37.5
43.75
50
56.25
62.5
68.75
75
81.25
87.5
93.75
100

Figure S2

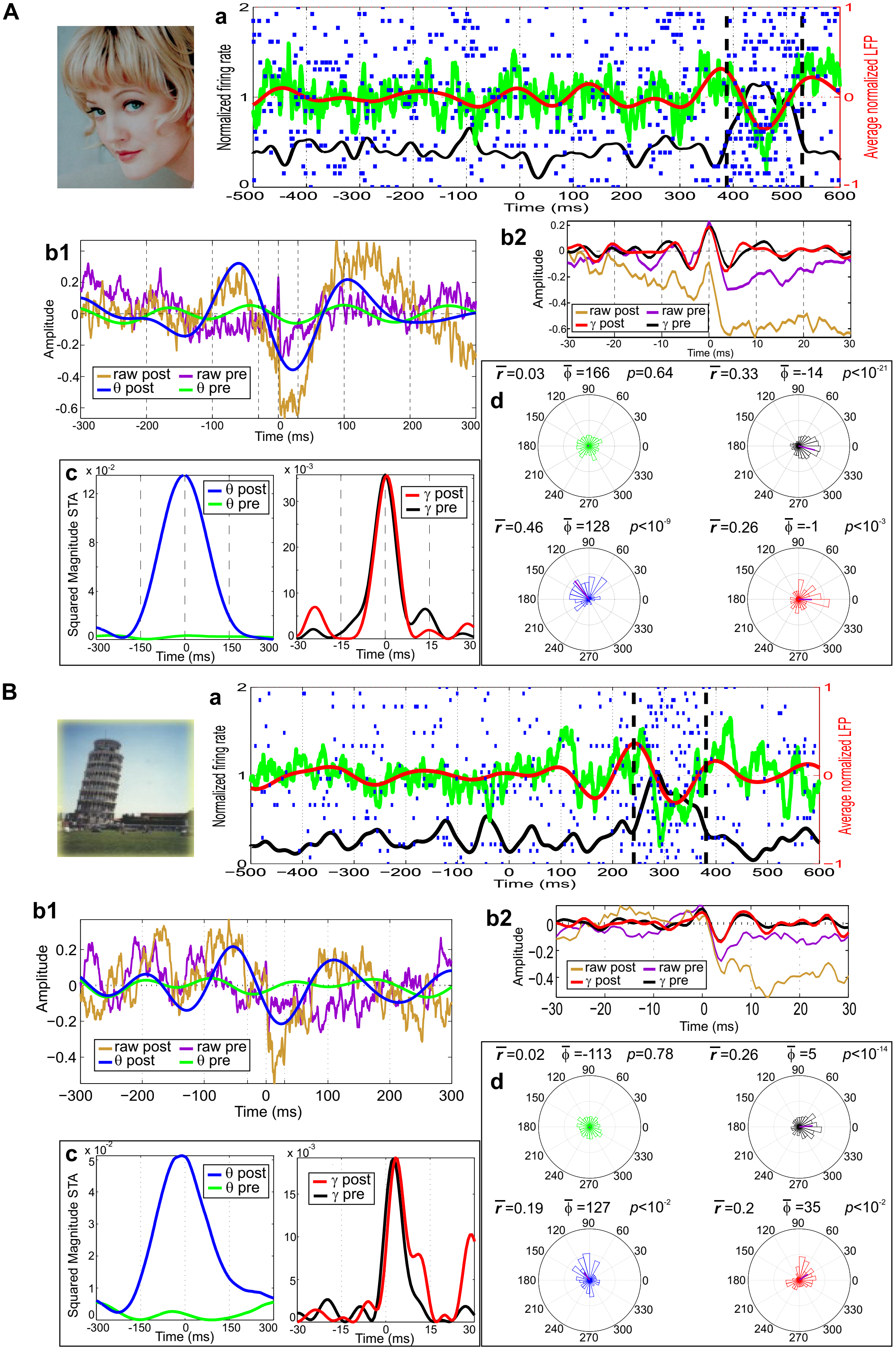
A
a
Normalized firing rate
Average normalized LFP
Time (ms)
b1
Amplitude
raw post
raw pre
θ post
θ pre
Time (ms)
b2
Amplitude
raw post
raw pre
γ post
γ pre
Time (ms)
c
Squared Magnitude STA
x 10-2
x 10-3
θ post
θ pre
γ post
γ pre
Time (ms)
d
r̄=0.03 φ̄=166 p=0.64
r̄=0.33 φ̄=-14 p<10-21
r̄=0.46 φ̄=128 p<10-9
r̄=0.26 φ̄=-1 p<10-3
B
a
Normalized firing rate
Average normalized LFP
Time (ms)
b1
Amplitude
raw post
raw pre
θ post
θ pre
Time (ms)
b2
Amplitude
raw post
raw pre
γ post
γ pre
Time (ms)
c
Squared Magnitude STA
x 10-2
x 10-3
θ post
θ pre
γ post
γ pre
Time (ms)
d
r̄=0.02 φ̄=-113 p=0.78
r̄=0.26 φ̄=5 p<10-14
r̄=0.19 φ̄=127 p<10-2
r̄=0.2 φ̄=35 p<10-2

Figure S3

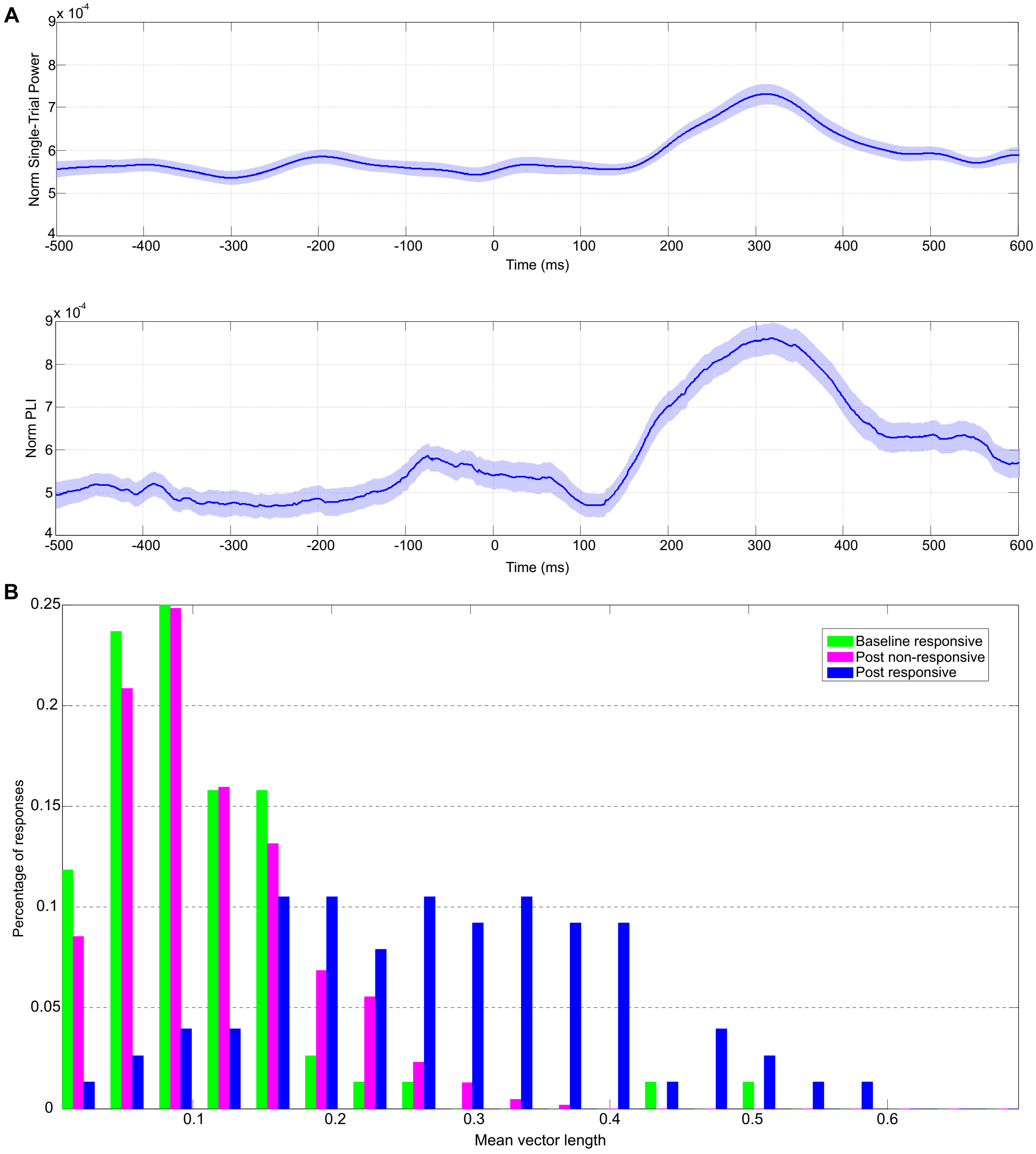
A
x 10^-4
Norm Single-Trial Power
Time (ms)
Norm PLI
B
Percentage of responses
Mean vector length
Baseline responsive
Post non-responsive
Post responsive

Figure S4

Figure S1, related to Figure 1. Behavioral results (based on all trials, regardless of the spike or LFP responses) (**A**) Histogram of the number of recognized trials for each stimulus used in the task (mean and median were equal to 28, whereas the standard deviation was 2.9). The maximum possible number was 32 (8 repetitions at 4 different durations) and we discarded all the stimuli (7%) that lead to less than 20 recognized trials, in order to be able to compute reliable LFPs. (**B**) Mean percentage of recognized trials grouped by stimulus duration. There is a large increase in performance going from 33 to 67 ms durations. Error bars denote SEM. (**C**) Percentage of recognized trials as a function of trial number for all durations. There is a significant priming effect (non-recognized trials tend to be the first ones in the sequence) for the 33, 67 and 100 ms presentations (p=0.02, $p<10^{-5}$, $p<10^{-10}$, respectively) but not for the 250 ms presentation (p=0.46). χ^2-test of homogeneity.

Figure S2, related to Figure 2. Selectivity of the spiking responses. (**A**) Histogram of stimulus selectivity. For each of the responsive units we computed the percentage of stimuli that elicited a spiking response. Since there were a maximum of 16 stimuli in a session, the minimum possible value was 1/16=0.0625. However, these stimuli were chosen from screening sessions were ~100 pictures were shown, and it has been reported [S1] that spiking responses in the MTL are very selective, with units responding on average only to 2-3% of the presented pictures. The arrows point to the selectivity value associated to exemplary responses shown in the next panels. (**B**) - (**C**) Responses (raster plots) from single units to all pictures presented in the session. Blue trials were recognized, whereas red ones were not. (**B**) Single unit from the right hippocampus showing a significant response only to the picture of Whoopi Goldberg. (**C**) A less selective single unit from the right entorhinal cortex showing significant responses to the pictures of Jerry Seinfeld, Michael Richards, Lisa Kudrow and Jennifer Aniston.

Figure S3, related to Figure 3. Example of responsive neurons. (**A**) Example from the right entorhinal cortex showing a spiking response to a picture of Drew Barrymore. (**a**) Each row in the raster plot is associated to one of the 27 recognized trials. Instantaneous firing rate (black solid); onset and offset of the spiking response (dashed lines); average LFP between 2 and 512 Hz (green); average LFP in the theta band (4-8 Hz; red). (**b**) Grand average of the STAs based on the spikes from pre- and post-stimulus epochs. Raw and theta-filtered data are shown in (**b1**), while raw and gamma-filtered data appear on (**b2**). (**c**) Grand average of the squared magnitude envelope of the STAs computed in the same epochs and bands as in (**b**). (**d**) Histograms of the spike phases on each epoch and frequency band. The resulting mean phase vectors (with magnitude $\bar{r}$ and angle $\bar{\phi}$) appear in purple, as well as the p-value from a Rayleigh test analyzing the uniformity of the phase distribution. (**B**) Another example, this time from a neuron from the left entorhinal cortex showing a spiking response to a picture of the Tower of Pisa. There were 28 recognized trials.

Figure S4, related to Figure 4. Phase resetting/locking analysis. (**A**) The single trial LFPs were filtered in the theta band (between 4 and 8 Hz) for the 76 significant responses, and the instantaneous power and phase were computed using the Hilbert transform. Then, we calculated the time-resolved (single-trial) theta-power and the Phase Locking Index (PLI), as in previous works [S2]. Before computing the grand average, the power and PLI for each response was normalized by the area below the curve (as we did in **Figure 3A**). *Top*, Grand average power. *Bottom*, Grand average PLI. Although we observed an increase in PLI around 150 ms after stimulus onset, there was also an increase in the single-trial theta power at about the same time. Therefore, although we cannot completely rule out a phase resetting of ongoing oscillations, the observed evoked theta LFP responses was not solely due to phase resetting, as the single trial theta power also showed the presence of an additive response. (**B**) Distribution of phase locking values in the baseline and response periods for responsive units (same as in **Figure 4A**), and in a post-stimulus period (from

100 to 700 ms after stimulus onset) for the non-responsive units (we considered 1079 "non-responses" in which we got a reasonable number of spikes, more than 20, as for the responsive units). As can be observed, phase locking values for the non-responsive units are clearly lower than the ones obtained for the responsive ones (in the response period) and similar to the ones obtained in baseline.

SUPPLEMENTAL EXPERIMENTAL PROCEDURES

Subjects and recordings

Recordings came from 12 sessions in 5 patients with pharmacologically intractable epilepsy (all right-handed, two males, three females, 19 to 39 years old). Patients were implanted with chronic depth electrodes for 7–10 days to determine the seizure focus for possible surgical resection [S3]. All patients gave their written informed consent to participate in this study, which conformed to the guidelines of the Medical Institutional Review Board at University of California at Los Angeles. The electrode locations were based exclusively on clinical criteria and were verified by MRI or by CT co-registered to preoperative MRI. Each electrode probe had a total of nine microwires at its end, eight active recording channels and one (low impedance) reference. A scalp electrode was used as a ground for the recording system. The differential signal from the microwires was amplified by using a 64-channel Neuralynx system, filtered between 1 and 9000 Hz and sampled at 28 kHz. Data was recorded from hippocampus (8 probes), amygdala (5 probes) and entorhinal cortex (5 probes).

Subjects sat in bed facing a laptop computer with a 60-Hz refreshing rate monitor, where 16 pictures (including faces, animals and places) were used on each session, except for 2 sessions where 8 pictures were used. Each picture was shown 8 times in pseudo-random order at four different nominal durations: 33 ms, 67 ms, 100 ms, and 250 ms (2, 4, 6, and 15 refresh screens, respectively). The actual duration of the stimulus on the screen showed some variability (of a few milliseconds), due to inaccuracies in the presentation times consistent with reports from previous studies [S4,S5]. However, it has been shown that stimulus duration (and any stimulus duration variability) does not have much an effect on the MTL spike responses and the main factor determining their magnitude is whether the stimulus is recognized or not [19]. Further support to this claim is given by the fact that the recognized trials at the 33 ms nominal duration showed an evoked potential in the theta band, unlike the non-recognized trials at larger durations (i.e. at all but the 33 ms condition). The pictures used were selected based on screening sessions conducted prior to the experiment, in which a relatively large set of images (83 to 99) of persons, landmarks and animals were shown for 1 s to the patient, six times each in pseudo-random order (see [15] for details). Pictures eliciting the largest responses in any neuron were later used in the experiment at all four durations. Before the experiments, the subjects confirmed they recognized the pictures used. Picture presentations were immediately followed by a mask lasting 467 ms, 433 ms, 400 ms, and 250 ms, respectively (i.e., the duration of each picture presentation with mask was 500 ms). The mask was used to block retinal persistence of the images and it was generated with randomly shuffled pieces taken from different images. It was the same for all pictures to avoid giving extra information about the picture shown (i.e., that the patient could associate a picture with its corresponding mask, because of the relatively low number of pictures used). The images were displayed at the center of the screen and covered ≈1.5° of visual angle. Subjects triggered each picture presentations by pressing a key and were instructed to pay attention to the presented picture and then, after the mask went off the screen, respond whether or not they recognized the specific person, animal, or landmark displayed (e.g., Julia Roberts, an eagle, Tower of Pisa) by pressing the left and right arrow keys, respectively. Figure S1 shows results of the behavioral performance during the task.

With the same experimental data employed in [19], where only spiking activity was analyzed, we here study the LFP responses and their correlation with the neurons' firing. In that first study, it was shown that MTL selective spiking activity appeared only for recognized trials. Moreover, the spiking responses were essentially all-or-none with only marginal changes with stimulus duration. Therefore, we collapsed the data from the different durations, meaning that for each stimulus we ended up with 32 trials, to study LFP responses and how they correlated with the neuron's firing.

Local Field Potentials

From the raw data, we removed all the detected spikes by linearly interpolating the signal from 1 ms before to 2 ms after the spike peak. The resulting signal was filtered between 2 and 512 Hz (zero-phase elliptic filter). A notch filter (2nd order IIR) was used to remove 60 Hz line noise and its harmonics. Finally, the signal was downsampled from 28 kHz to 1.5 kHz. We computed the power spectrum for each of the 328 recorded channels and discarded those exhibiting very large high frequency noise. Overall we discarded 5 channels from hippocampus, 15 from amygdala, and 7 from entorhinal cortex; 8% in total), resulting in a final set of 301 channels.

The single-trial LFP traces were extracted from 1 s before to 1.2 s after stimulus onset. For each channel, the mean and standard deviation at each sample point were calculated across all trials and we automatically discarded trials having high amplitude artifacts, i.e. those with more than 1% of the samples outside of the mean ± 5 standard deviation range. The remaining trials were normalized by the mean and standard deviation during the baseline period (-500 to -100 ms from stimulus onset). To have sufficient statistical power, we excluded from the analyses responses with less than 20 recognized trials.

Single unit responses

Spike detection and sorting was done with Wave_Clus [S6]. From the 301 analyzed channels, 52% showed spiking activity (either multi- or single-unit). For each stimulus, we split the trials into recognized (rec) and non-recognized (nonrec) based on the behavioral responses. Given that MTL neurons tend to respond only to recognized pictures [19], the post-stimulus response to a picture was defined as the median number of spikes across rec trials only, between 200 and 600 ms after stimulus onset. The baseline for each picture was the median number of spikes across all trials (rec + nonrec), between 500 and 100 ms before stimulus onset. We considered a unit response to be significant if it fulfilled four criteria: (i) the post stimulus response was larger than the mean baseline plus 4 standard deviations (across all stimuli), (ii) the median number of post stimulus spikes was at least 2, (iii) the *p*-value of a ranksum test between the spike count on each trial for the post stimulus and baseline (for the particular picture) was less than 0.01, and (iv) the instantaneous firing rate had to cross over a threshold for at least 85 ms (with the upwards crossing defining the spiking response onset). The instantaneous firing rate was calculated by convolving the spike counts in bins of width 0.65 ms (one over the sampling rate) with a Gaussian kernel with σ=10 ms (truncated at 1% amplitude). The threshold was set to the mean + 5 s. d., computed across all stimuli for the baseline period (with a minimum at 10 Hz, for neurons with low baseline firing). The combination of these criteria matched closely what was visually identified as significant responses (avoiding obvious false positives and misses), as in previous studies [15,17].

LFP power analysis

We performed a time-frequency decomposition of the LFPs using the continuous wavelet transform. For this, the signals were convolved with a complex Morlet wavelet defined as

$$\Psi(f_0,t)=\frac{1}{(\sigma^2\pi)^{1/4}}e^{-\frac{t^2}{2\sigma^2}}e^{2\pi i f_0 t}$$

This wavelet is a complex sine wave with central frequency f_0 and amplitude tapered by a Gaussian with standard deviation σ, truncated at the 1% level. The Morlet wavelet can be characterized by the *wave number* $n_c=2\pi\sigma f_0$, which was set to 4. This means that each wavelet had approximately 4 cycles, and, the larger the frequency f_0, the shorter the wavelet duration.

The wavelet transform gives a series of complex coefficients of the form

$$W(f_0,t) = A(f_0,t)e^{i\phi(f_0,t)}$$

For each frequency f_0 we extracted the instantaneous power $A^2(f_0,t)$, and to avoid redundancy we considered a logarithmic set of frequencies, starting at 2 Hz and increasing by $2^{1/4}$. Time-frequency plots were normalized at each scale by the mean power (at that scale) computed during the baseline period (from -500 to -100 ms).

In the cases with significant unit responses, we evaluated differences in the LFP power between the recognized (2054) and the non-recognized (288) trials in different regions of interest (ROI, marked with black rectangles in Figure 1). The baseline ROIs were defined using a window spanning the same frequency ranges and time-width of the ROIs defined after stimulus onset. For the single trial power analysis, we used a ranksum test to evaluate differences between the mean power of the single trial responses (comparison against baseline and across conditions), whereas for the evoked power, since a single average LFP can be obtained for a given number of trials, we tested the difference between the mean powers within each ROI using 1000 bootstrapped samples. Comparisons against baseline were done using percentile confidence intervals from the bootstrapped samples [S7]. To have a fair comparison of the activation in the ROIs after stimulus onset across conditions (recognized and non-recognized), we took samples of equal size (288 trials) for both conditions and performed a ranksum test.

In order to show the consistency of the results across subjects and responses, we also performed the same statistical analysis but eliminating the responses from one subject at a time. In addition, we used the average values on the ROIs of the 76 individual responses (i.e. grouping by response, instead of pooling from the whole set) for the recognized trials (the number of non-recognized trials was not large enough trials to do this analysis), and confirmed that results were very similar to those reported for the whole dataset.

LFP selectivity analysis

We studied the degree of spatial localization and stimulus specificity of the evoked-theta and single-trial gamma responses. For a given stimulus, we considered that a channel had a significant LFP (single trial) gamma response if there was a significant difference (p<0.05) between the (single trial) mean power in the gamma–ROI and the analogous one in the baseline period (ranksum test). Given that for the theta average responses we did not have single trial values, in this case we considered that a theta power increase was significant if the mean power (of the average LFP) in the theta–ROI was larger than $\mu_b + z_{0.995}\,\sigma_b$, where μ_b and σ_b are the mean and standard deviation of the mean power at the analogous ROI in the baseline period computed across all stimuli, and $z_{0.995}$ is a standard Gaussian percentile. To study the spatial distribution of the LFP responses, for those channels and stimuli were we observed a unit response we computed the probability of finding an LFP response (theta or gamma) in: 1) the same channel (RCh), 2) other nearby channels corresponding to the same intracranial probe (RPr), 3) channels from other more distant probes ("Other"), i.e., probes that had no channel with a spiking response to the stimulus. For these calculations, we eliminated probes with less than 4 available channels. For statistical comparisons among conditions 1 to 3 we used a ranksum test for RPr vs. "Other", and sign tests for RPr and "Other" against the pointwise estimates obtained for RCh.

To study the selectivity of the LFP responses to the different (recognized) stimuli, for each channel with unit responses to a given stimulus we computed the percentage of stimuli that elicited a response in terms of unit firing (see Figure S2 for the distribution across all spiking responses and the raster plots of two exemplary units for all the stimuli presented), theta power or (single trial) gamma power. In this case, statistical significance was assessed by using a non-parametric ANOVA (Kruskal-Wallis) followed by pairwise ranksum tests.

Instantaneous power, Spike Triggered Averages (STA), and phase locking

In those cases were we found significant theta responses in the same channels were we found unit responses, we calculated the instantaneous power in the theta band using the squared magnitude of the Hilbert transform of the average LFP filtered between 4 and 8 Hz. Similarly, we calculated the instantaneous (single trial) gamma power by averaging the single trial LFP responses filtered between 70 and 200 Hz. Each response (power or firing rate) was normalized by the total power (area below the curve) before computing the grand averages.

For the unit responses we considered the spikes in two epochs: “pre-stimulus” (baseline period between -500 and -100 ms) and “post-stimulus” (within 85 ms from the spiking response onset, see above). Cases with less than 20 spikes were discarded. We then computed the STA, i.e., the average of the LFP traces aligned to the time of the spikes. As different units may show phase locking at different phases, the locking effect might be reduced when averaging STAs from different channels. Therefore, we also computed the envelope of the STA using the squared magnitude of the Hilbert transform filtered in the theta and gamma bands. In this way we get a measure of how strong is the locking in the set of units analyzed.

For the phase locking analysis, in addition to the “post-stimulus” epoch we used a “pre-stimulus” epoch defined by an 85 ms window chosen in the baseline period. Given than in several cases there were too few spikes, in this case for the “pre-stimulus” epoch we considered the spikes from all the stimuli. At each spike time, we computed the phase using the angle of the Hilbert transform of the LFP filtered in the appropriate band (with 0° and ±180° representing the peak and trough of the oscillation, respectively). For each spiking response and epoch we computed the mean resultant vector (with its magnitude being the phase locking value and its angle the circular mean of all the angles in the window) and performed a Rayleigh test (evaluating the hypothesis of a uniform phase distribution).

In order to further explore the phase relationship, Figure S4A presents a phase resetting analysis. Although we cannot completely rule out a phase resetting of ongoing oscillations, the observed evoked theta LFP responses was not solely due to phase resetting, as the single trial theta power also showed the presence of an additive response. Moreover, Figure S4B shows that phase locking values computed for non-responsive units are clearly lower than the ones obtained for the responsive ones (in the response period) and similar to the ones obtained in baseline.

Supplemental References

S1. Quian Quiroga, R., Reddy, L., Koch, C., and Fried, I. (2007). Decoding visual inputs from multiple neurons in the human temporal lobe. J. Neurophysiol. *4,* 1997-2007.

S2. Tallon-Baudry, C., Bertrand, O., Delpuech, C., and Pernier, J. (1996). Stimulus specificity of phase-locked and non-phase-locked 40 Hz visual responses in human. The Journal of Neuroscience *13,* 4240-4249.

S3. Fried, I., MacDonald, K.A., and Wilson, C.L. (1997). Single neuron activity in human hippocampus and amygdala during recognition of faces and objects. Neuron *5,* 753-765.

S4. Wang, P., and Nikolić, D. (2011). An LCD monitor with sufficiently precise timing for research in vision. Frontiers in human neuroscience

S5. Wiens, S., Fransson, P., Dietrich, T., Lohmann, P., Ingvar, M., and Arne, Ö. (2004). Keeping It Short A Comparison of Methods for Brief Picture Presentation. Psychological Science *4,* 282-285.

S6. Quian Quiroga, R., Nadasdy, Z., and Ben-Shaul, Y. (2004). Unsupervised spike detection and sorting with wavelets and superparamagnetic clustering. Neural Comput. *8,* 1661-1687.

S7. Davison, A.C., and Hinkley, D.V. (1997). Bootstrap methods and their application Cambridge University press).
